# Antenatal depression in Sri Lanka: a qualitative study of public health midwives’ views and practices

**DOI:** 10.1186/s12978-022-01330-z

**Published:** 2022-01-28

**Authors:** Sage Wyatt, Truls Ostbye, Vijitha De Silva, Qian Long

**Affiliations:** 1 Global Health Research Center, Duke Kunshan University, No. 8 Duke Avenue, Kunshan, 215316 Jiangsu China; 2 Duke Global Health Institute, Duke University, Durham, NC USA; 3 University of Bergen, Bergen, Vestland, Norway; 4 Faculty of Medicine, University of Ruhuna, Matara, Southern Province Sri Lanka

**Keywords:** Depression diagnosis, Antenatal care, Mental health services, Sri Lanka, Health personnel

## Abstract

**Background:**

Almost all pregnant people in Sri Lanka receive antenatal care by public health midwives. While there is strong infrastructure in Sri Lanka for postpartum mental health care, the current practices within antenatal mental health care have not been externally evaluated. The purpose of this study is to investigate the current clinical guidelines and experiences of how public health midwives diagnose and treat antenatal depression.

**Methods:**

We conducted in-depth interviews with 12 public health midwives from four antenatal clinics in the Bope Poddala division in Galle, Sri Lanka and reviewed and extracted information on antenatal depression from clinical guidelines. Data was collected in Sinhala and translated into English. We used applied thematic analysis and worked closely with our local team to ensure data trustworthiness.

**Results:**

Midwives (n = 12) reported varying degrees of knowledge on antenatal depression and did not have standardized diagnosis patterns. However, they were very consistent in their clinical practices, following guidelines for referral and follow-up of case management, building strong rapport. In their practice, midwives continue to face challenges of lack of human resources and high stigma around mental illness. They suggested that that care could be improved with use of a standardized diagnostic tool, and easier access to specialist care. We found the clinical guideline on the diagnosis and treatment of antenatal depression is lacking key details on symptoms for appropriate diagnosis, but it clearly guides on how to navigate treatment.

**Conclusions:**

Public health midwives are following the clinical guideline to refer pregnant women who need intervention for antenatal depression and follow-up for case management. However, there is a need for more specific and context-relevant guidelines, especially for diagnosis of antenatal depression. Formative research is needed to explore intervention strategies to improve antenatal depression management in Sri Lanka.

**Supplementary Information:**

The online version contains supplementary material available at 10.1186/s12978-022-01330-z.

## Plain language summary

We interviewed 12 midwives at pregnancy clinics in southern Sri Lanka about what happens if a pregnant woman gets depressed. Some midwives knew a lot about depression, while others did not know very much. They all had different ideas of what the signs of depression were, and what percentage of pregnant women are usually depressed. They have a rule book about how to be a midwife, but it is not clear about how they should find out if a pregnant woman has depression. However, all midwives agreed on what they should do if they meet a depressed woman during an antenatal appointment in their clinic, following the rule book closely. They said they need to tell their supervisor, who will tell a psychiatrist who can treat the depression. However, sometimes there are problems. Midwives said that they, their supervisor and the psychiatrist are all very busy and don’t have enough time to spend with pregnant women. Also, a lot of the women in their clinics don’t want to get treated because they feel embarrassed about having depression, and don’t want other people to know. Midwives told us they could do their jobs better if they could give a quick test for depression to every woman visiting their clinic. This would be an easy solution, because they already use a test like this for after women give birth.

**Supplementary Information:**

The online version contains supplementary material available at 10.1186/s12978-022-01330-z.

## Background

Antenatal depression is defined by depressive symptoms in pregnant women. This condition can have negative effects on the health of both mother and fetus, and it is estimated that half of all postpartum depression cases begin during pregnancy. In low and middle income countries globally the estimated prevalence of antenatal depression is between 5–50%. However, detection of the condition remains a challenge, as symptoms vary across trimester and many signs of depression overlap with somatic effects of pregnancy, such as fatigue [4, 7].

Internationally, screening tools are widely used to assess for antenatal depression. These tools are not meant to diagnose depression but to alert healthcare providers to symptoms which will require further assessment. The Edinburgh Postpartum Depression scale is a very widely validated questionnaire for both antenatal and postnatal depression, advantageous for excluding somatic symptoms of normal pregnancy [4, 23]. An actual clinical diagnosis should be made by a mental healthcare provider. Despite some research indicating that medications used to treat depression may pose risks to fetal health, they are still recommended for use during pregnancy considering that depression itself also holds risk for the fetus. Any other evidence-based treatments used for major depression are also recommended during the antenatal period [11].

Despite LMICs showing a higher prevalence of antenatal depression on average compared to high income countries, they are host to less than 20% of the world’s mental health resources [7, 31]. Mental healthcare systems with more limited resources in low and middle income countries face many additional challenges in the diagnosis and treatment of antenatal depression, including insufficient funding, sparse mental healthcare specialists, poor access and education, and lack of clear policy guidelines [28]. While the need for improved mental healthcare in settings with limited resources has been widely recognized for many years, international standard guidelines have not been entirely effective without context specific adaptation [8].

Sri Lanka, despite being categorized as a low and middle income country and reporting relatively low economic indicators, has a very strong healthcare infrastructure, especially for maternal and child health. Nearly all pregnant women nationally receive antenatal care and skilled birth attendance, with almost nonexistent income disparity. Each clinic is supervised by a medical officer of health (MOH), but much of the frontline care is done by Public Health Midwives (PHMs). There is an organized referral system for pregnant women who require specialized care beyond what PHMs are trained to provide. There has been a national mental healthcare policy since 2005, including many types of providers and both inpatient and outpatient treatment facilities. On a district level, care is directed by a medical officer of mental health who provides home visits or sees patients in an outpatient clinic [26].

However, literature regarding the state of antenatal mental healthcare in Sri Lanka is still extremely limited, despite evidence of high prevalence of antenatal depression at 16.2%, with 0.8% expressing suicidal ideation [1, 6]. Suicide in 2013 was identified as the most common cause of maternal mortality in Sri Lanka, yet there has still there has been limited research investigating how the antenatal mental health system works and how it should be reformed to prevent these deaths in the future [2]. The purpose of this study is to describe the current policies surrounding mental healthcare in an antenatal care setting, and to compare these policies to real-world community practices.

## Methods

### Setting

We performed a qualitative study in the Bope-Poddala MOH division of Galle district, Sri Lanka. During the time of data collection, the Bope-Poddala division held four field clinics once every 2 weeks to deliver maternal and child health services to the community: Kuruduwatta clinic, Ukwatta clinic, Meepawala clinic, and Bope clinic. Each clinic hosts one medical doctor (MOH) and four to five public health midwives in charge of antenatal care. Demographics of each clinic varied, as Ukwatta and Kuruduwata clinics serve a majority Muslim population, while Meepawala and Bope clinics serve a majority Sinhalese Buddhist population. As Muslims are considered an ethnic and religious minority in Sri Lanka, this community is an outlier demographically.

### Standard treatment protocol review

When we conducted the in-depth interviews in the study site, the Sri Lankan author (VDS) consulted with the MOH in Bope Poddala about what kinds of guides or policies dictate treatment practices in antenatal clinics. This physician recommended we use the clinical guidelines for maternal health, which is a nation-wide clinical protocol to standardize care across all facilities. The title of this document is “Maternal care package: a guide to field healthcare workers.” We located an online, English-translation copy at https://medicine.kln.ac.lk/depts/publichealth/Fixed_Learning/clearkship/3.PHM/maternal_care_package_a_guide_to_field_healthcare_workers_english.pdf [15].

We used the document as a benchmark for ideal antenatal care and to provide a contextual reference for the data collected from midwives. To identify passages of the texts relating to antenatal mental healthcare, we conducted an electronic key word search in English for relevant phrases to our research question: “depression”, “mental” and “psych”. We excluded sections on the postpartum period, as practices for post-partum mental healthcare are very explicit and well-documented. Search results that were not relevant, such as detecting “mental” as a part of another word (e.g. “instrumental”) were not included. Instructions on mental health for the baby were also not included. The extracted data was recorded electronically in the form of a table included in Additional file 1.

### In-depths interviews

We conducted in-depth interviews from June to October 2020. I (SW) developed a semi-structured interview guide in English including three exploratory themes: knowledge of antenatal depression, mental health care practices at the clinic, and suggestions for improvement in protocol or policy. The content of the guide was informed by existing literature and discussed and reviewed by the research team. The guide was translated into Sinhala by the moderators before the interviews, and pretested during mock interviews.

The author located in Sri Lanka (VDS) recruited 2 physicians who graduated from medical school but not had yet finished their internship training to conduct qualitative interviews at the study site. They were experienced in conducting in-depth interviews and proficient in both Sinhala and English. In addition to their prior experience with projects at the University of Ruhuna and qualitative research coursework during their medical studies, the interviewers also performed two mock interviews, as training, before data collection began. All interviews were conducted in Sinhala, typically lasting approximately 1 hour. They conducted semi-structured interviews with 12 PHMs from the four public clinics in the Bope-Poddala MOH division. Any midwife currently employed at one of these clinics was eligible for participation. Participant selection was performed by convenience sampling, with any midwife not currently busy asked to join the study and interviewed after their shift ended. During the data collection period, there were a total of 18 PHMs in the MOH division, and 6 were not selected due to unavailability or lack of interest. All participating midwives were given a small cash gift of 1000 Rs (approximately 5 USD) for their time.

The interviews were audio recorded. Recordings were transcribed directly into English while listening to the Sinhala recording by the moderator who performed the interviews. The moderator then wrote a summary of each transcript in addition to the verbatim translation. The second moderator then verified the translation by reading the transcript and listening to the recording simultaneously. English transcripts and summaries were then shared by a secure REDcap network with the research team for further analysis.

I (SW) led the data analysis with input from all team members. My original research plan included a 3 month stay in Sri Lanka to manage data collection, observe antenatal care clinics, and collect non-verbal data. However, these travel plans were cancelled due to the COVID-19 pandemic. Therefore, I worked closely with the local team led by my supervising professor (VDS) to manage data collection and gain a good understanding of the context and data interpretation. However, some procedures that could have added to the confirmability and authenticity of the study are limited, such as non-verbal data and an iterative development of the topic guide.

Coding was guided by the theories of applied thematic analysis [14], coded in NVivo 12. I (SW) read the transcripts line by line to identify key concepts and develop a coding scheme, discussing the process with the team member most experienced in qualitative research (QL). Structural and emergent codes were identified. This coding scheme was further refined after memo writing on uncoded data, preliminarily coded data, and re-coded data. Results were regularly discussed with the team member (VDS) who is a physician-scientist in Southern Province, Sri Lanka, and later reanalyzed after his feedback. I also consulted the summaries written by the moderators including their interpretations of the data to clarify any portions difficult to understand.

## Results

### Standard treatment protocol review

The clinical treatment protocol outlined recommended practices for mental healthcare at both clinic and home visits. It listed depression and mental disorders generally as important health risks during pregnancy that must be treated, similar to other physical health risks. It was recommended that mental health status be assessed by observing changes to behavior and reporting social risk factors during intake, as well as asking women about their mental wellbeing directly. It is emphasized in the initial intake form that PHMs are responsible for pregnant women’s whole health, physical, psychological, and social.

For treatment of “mental disorders,” PHMs were guided to refer mothers they judge to be at risk to their supervising obstetrician (MOH). The recommended frequency of home visits is also increased from once per trimester to once per month. They have additional duties to check compliance of drugs that the mothers in their care will be given after a referral to a psychiatrist. There is an additional note that it is the PHM’s responsibility to educate the family members on mental disorders, when to seek further help, the importance of taking the prescribed medication, and the high risk of postpartum depression after antenatal depression.

### In-depth interviews

#### Participant characteristics

The 12 participants in the sample were all actively working as public health midwives at the time of the interview. All participants were ethnically and religiously Sinhalese Buddhists, and all were women. There were 3 participants who had one child, and 9 (75%) were multiparous. They varied in age from 35 to 64 years old. The length of time they had worked as a midwife ranged from 6 to 30 years, while the time of working at the particular clinic where each midwife was interviewed varied from 2 to 30 years. The majority (83%) had worked at their current clinic for less than or equal to half of their career.

#### Typical practices

##### Diagnostic process

Perceived prevalence of antenatal depression varied greatly across the participants. Seven participants said that antenatal depression is rare, and they have only met a handful across their careers. The other 5 participants reported that mental health problems are common among pregnant women in their care, and they treat such mothers on a regular basis. Of the 8 midwives who were asked, 4 said they believed mental health during pregnancy is an important issue, and 4 said they did not think so.

All PHMs described themselves as the frontline care workers for mental health, being the primary stakeholder in identification of antenatal depression. Currently, there is no screening tool used for women in the antenatal period, and midwives’ knowledge is based solely on their own observations. Some midwives cited information collected about the family history of mental health as a kind of screening process. However, the initial diagnosis of depression is most often determined subjectively through midwives’ direct observation of mothers’ behavior or speech during antenatal care visits.

Honestly speaking we do not have a proper way to identify antenatal mental health issues. If we notice any differences in their behavior only we pay special attention and identify their problem. Apart from this on the booking visit we get a brief history on personal or family history of mental health issues and pay special attention to them if there is a strong family history or personal history of mental health issues.—PHM 12

When asked about the symptoms of depression, all midwives said that depression can be identified by talking with and observing the mothers in their care. Descriptions of typical symptoms were varied, for example some said they are alerted to a problem when mothers are overtalkative and asking too many questions, while others described mothers who are withdrawn and untalkative as being at risk for depression. There was a consensus that depressed mothers had mood changes, poor concentration, or seemed to be overthinking. When making home visits, midwives said they saw a disorderly home environment and poor personal hygiene of the pregnant woman to be signs of mental health problems.

We identify such mothers usually when we talk with them. These mothers usually don’t maintain eye contact when they talk with us. Some do not pay attention to what we say, do not seem to understand us and respond poorly while some others seem to be overthinking. When we see these features in mothers we tend to pay more visits and dig to look for any mental health issues that they may be suffering from.—PHM 1

Sometimes we note that they are less active than earlier. Sometimes they are not attending to household works like earlier. And sometimes they pay less attention to their personal hygiene.—PHM 6

There are also instances where pregnant women themselves seek help from midwives about symptoms of depression, though lay women do not usually identify the problem as a mental health issue directly.

Some of them directly come to us complaining about poor appetite and insomnia. I have also met mothers with mental health problems who easily burst into tears and multiparous mothers who excessively complain about their kids being disobedient and troublesome, but later come to find that ‘The Issue’ is elsewhere, in the woman’s mind.—PHM 1

Talking with mothers’ family members about their observations of the mother’s behavior was also identified as an important part of the diagnostic process, as the family often notices behavioral changes before midwives can. Diagnosis of depression can come as a comfort to the family, who are given an explanation for why the pregnant woman’s mood has changed and an actionable solution for recovery.

By the time the diagnosis is revealed, the family members have some idea that the pregnant mother is suffering from some kind of psychiatric illness. So, they do not deny but prefer to keep it a secret from outsiders (neighbours etc.). And when the mother shows symptoms like short temper, forgetfulness etc., the family is more tolerant as they know that she’s not in a sound mind.—PHM 11

Most of the time family members identify alterations in their behavior before health staff do. Most of the time they pay attention to these symptoms and seek medical attention. – PHM 10

##### Referral and follow-up process

After these initial investigations, PHMs start the referral process, which had very consistent descriptions across all participants. The referral process begins with the midwife, who sends pregnant women to meet with the clinic physician and then the district psychiatrist. The psychiatrist provides treatment, and follow-up is monitored again by the midwives.

When we meet a mother whom we suspect to have a mental health issue, we refer them to the MOH and he refers the patient to the psychiatrist assigned to our area. Then she sees the pregnant mother with the mental health issue at the clinic or at Karapitiya hospital. After treating her she is followed up both at the clinic and the hospital and also we do home visits.—PHM 4

The midwives aid in the treatment process, communicating with the psychiatrist and monitoring pregnant women. They act as the primary stakeholder in treatment follow-up, continuing home visits after treatment initiation to check on the progress of the mothers in their care.

There is a book in the clinic premises to record if there are mothers with mental health issues. There, the psychiatrist writes notes and records information that we as midwives need to know about the patient. For instance, she makes a note there if she needs us to pay more frequent home visits.—PHM 11

Generally, most of the participants assured that treatment compliance in their communities is satisfactory, especially with strong family support and the extra care they provide during their practice with follow-up visits. Midwives considered an important part of their duty to be serving as an educator to help the family understand more about antenatal depression and gain their support for the treatment process.

We as midwives do more frequent home visits to ensure her compliance, to make sure she takes her medications at the correct time, to check her improvement and to educate the family members and gain their support. We also visit them along with the psychiatrist when we check her improvement to decide on further treatments.—PHM 3

We talk with the patient as well as with the family members spending a lot of time with them. We advise the family on how to take care of a mother with such a problem. We explain to them the importance of their support in managing the mental health issues in these mothers… If mothers are not willing to take medications, we give the responsibility to one of the caretakers to give the medicines on time.—PHM 6

We specifically take effort to explain the pregnant mother’s condition to the family, during our home visits, because it’s essential to obtain their support for the process of treatment as well as to make home environment favorable for improvement of the mother.—PHM 1

PHMs generally felt satisfied with the quality of their work and perceived that the mothers appreciate the mental health services they provide.

And when they feel well, they are very much grateful that we could identify and direct her to medical attention, when even she herself had no clue of an illness.—PHM 8

Rather than a service they perceive it as help that we do for a friend, because we get close with them when discussing their problems.—PHM 1

#### Challenges and midwives’ suggestions for change

##### Diagnostic process

Some challenges do occur after treatment has been initiated. While consistently participants reported that the majority of families are supportive, bad reactions do occur. Some families initially do not want to accept the diagnosis or treat the pregnant woman poorly due to stigma and misconceptions about mental illness. Then, while families are willing to discuss the issue with health care providers, they try to keep it a secret from others in the community.

When the pregnant woman is identified as having a psychiatric illness/problem, the family tends to label her as a ‘psychiatric patient’ and make her feel left out. Then, although she is successfully medically treated the improvement is limited because of insulting remarks and discouragement from the family.—PHM 1

There are instances where they do not accept the diagnosis, and sometimes they complain that she is pretending these symptoms.—PHM 5

They do not want the neighbors to get to know that there’s a psychiatric patient in the family. Therefore, there’s a tendency of hiding the symptoms by the family but aren’t reluctant to reveal us.—PHM 3

Many midwives gave specific suggestions for improving the diagnostic process. There was strong agreement among the participants that the referral to the psychiatrist must be related to the mothers in a sensitive manner, due to heavy stigma surrounding mental illness. Most believed it is better to explain the condition in vague terms and conceal some information during the referral process.

We usually don’t tell the mother directly that she’s been diagnosed with a psychiatric illness. Even when referring her to the psychiatrist at MOH we do not tell that she’s a doctor for patients with psychiatric illnesses. We reveal the diagnosis only in unavoidable situations. The majority do not refuse the treatments.—PHM 12

Several PHMs suggested that using the same screening test for antenatal depression as is currently used for postpartum depression because this would help detect mental health problems at an earlier stage, averting crisis situations.

It would be good if we had a screening test like in postpartum depression. For an example if we arrange a time in each trimester and do a screening test we can pick these mothers efficiently.—PHM 2.

More educational programs could also reduce stigma overall and might increase self-reporting of mental health issues. To prevent depression during pregnancy, they also suggested that programs such as domestic abuse hotlines be put in place to prevent the root causes of mental health problems.

If the awareness in the community can be improved through lectures/posters or by using new technology such as social media it would be of great help to identify mothers with mental health issues earlier, because then they themselves are aware of the symptoms or at least a family member may notice and bring her to medical attention early. When they have a good understanding, they won’t try to hide themselves or a family member and won’t be reluctant to accept treatments… Rules and regulations regarding abuse of women, should be tightened and there should be an easily approachable service to gain help (legal, mental etc.) for victimized women because ‘abuse’ is a major cause that leads to depression.—PHM 9

##### Treatment process

Midwives also reported some difficulties during the treatment and referral process. To see the psychiatrist, PHMs said mothers have the option to travel to a public referral hospital or see the psychiatrist when she comes to the local clinic a few times per month. The referral hospital can be a difficult environment for pregnant women because of high patient numbers and the strong stigma associated with the mental health ward.

The pregnant mothers that we refer are reluctant to attend the clinic at Karapitiya Teaching Hospital because they get more anxious and agitated seeing worse patients there and by receiving their inputs… Also mothers complain that the staff there is not patient friendly and a nursing officer had questioned a patient why she was sent there because that particular mother’s issue was not that apparent. Maybe the staff there is also under stress due to their workload. If improvements could be made to increase the staff or to limit the number of patients being seen by the psychiatrist per day, it would be convenient for patients as well as the staff.—PHM 6

Midwives suggested that changing the role of the psychiatrist to be more involved in the smaller antenatal care clinics rather than only at the district hospital would improve patient experience and increase diagnostic sensitivity. They suggested that if more psychiatrists could be hired to work in the area, care quality would benefit because they could have a greater involvement on a local level and provide more accessible services.

I think if our psychiatrist was more available for us to reach, it would be easier for the patients, as well as for us, because then there’s less chance that we might miss a patient. Also, if she herself can visit patients with poor family support and financial difficulties who are reluctant to visit MOH clinic or the hospital for consultations, it would be of much help to these poor mothers.—PHM 8

Lack of time for quality care was consistently discussed, especially as a barrier to treatment as mentioned before. Participants suggested that the district should recruit more midwives to reduce workload or reduce the number of mothers assigned to each midwife. Reducing workload would be beneficial to the pregnant women by improving quality of care and allowing for more home visits.

The only thing is we are overloaded with work, the population allocated to us is very large, therefore we face difficulties in giving lot of time to one mother… The government should allocate more human resources for our staff, so that these patients can be closely followed up and directed to proper treatment.—PHM 7

Generally, despite these issues, midwives believed that the current system is functioning, and further changes should aim to refine their current practices.

Rather than implementing new action plans I think it would be more important to make improvements in the current process.—PHM 1

## Discussion

The midwives had a variable understanding of the prevalence and symptoms of antenatal depression, which is partly attributable to limited clinical guidance for detection and management of mental health issues during the antenatal period. A previous paper published using quantitative data collected during this study identified that midwives also have heterogenous perspectives on risk factors for depressive symptoms [35]. Lack of knowledge about antenatal mental health among midwives in other contexts is not necessarily due to a lack of interest from the midwives but instead a lack of training resources [9]. Midwives are nonetheless very confident in the treatment protocol they follow, and act as key stakeholders connecting the pregnant women, the psychiatrist, and the pregnant women’s family for quality care. During this process, midwives also face additional challenges of frustration due to their increased workload and poor communication with the psychiatrist, as well as noncompliance of the mothers due to stigma surrounding mental health and poor hospital conditions. High workload, poor relationships with supervisors, and perceived lack of time to provide high quality care for patients are all risk factors for burnout among midwives worldwide [3]. However, in this study, midwives reported they are satisfied with the current system, and mostly made minor suggestions for improvement, such as integrating a depression screening questionnaire to the antenatal care protocol.

Data from this study showed that the current antenatal care treatment protocol includes very little information on the diagnosis of antenatal depression. The guidelines lack descriptions of specific symptomatology of depression, and instead focus on assessing practical impairment, such as hygiene and sleep patterns, which in theory is more practical for non-specialist healthcare workers to assess [20]. However, in practice midwives still assessed mental health status based on symptomatic indication, and lack of symptom-based diagnostic guidelines has translated into wide variation in perceived prevalence. Additionally, previous reports have shown that this protocol is not widely available in all clinics, and it was not clear if midwives in the Bope Poddala district regularly refer to it [26].

Previous studies have emphasized a process of non-specialist or community healthcare workers identifying mental illness at a community level as a key system to the future of mental healthcare in low and middle income countries [22]. An easy intervention to standardize this process in this setting, as suggested by our participants in this study, would be to use a screening questionnaire in addition to subjective observation. The suggestion is practical in a clinical context because, as one participant explicitly mentioned, the EPDS questionnaire is already used in clinics for postpartum depression and has already been validated for use antenatally in Sri Lanka [29].

The role of PHMs in this community encompasses many roles besides diagnosis, as they function as key stakeholders connecting the mother, the family, and the specialist. The importance of family was a consistent theme throughout the treatment process, as while some families can treat pregnant women poorly others can ensure a strong support system for the success of treatment. Patient and family centered care has demonstrated increased quality of care in other settings outside of Sri Lanka [25]. Evidence for increased quality through patient and family collaboration shown in this study is midwives’ perception of mothers’ satisfaction, and their perception that their relationship with the mother is on a more personal than professional level. It is this personal connection that allows midwives to overcome the issue of taboo for the end goal of mothers’ welfare, and Sri Lankan PHMs have been identified as key stakeholders for providing interventions for other highly taboo issues [16].

Despite this, directly asking mothers questions on their mental health as the official protocol suggests may be an impractical practice within this context. Mothers may attempt to hide their symptoms due to fear and stigma or consider them irrelevant to medical practice due to lack of knowledge [30]. A previous study in this community showed that for post-partum depression, new mothers are very unwilling to seek care for psychiatric illness, and can be discouraged further by their family members [13]. Midwives in this community may turn to concealing the diagnosis from the mother in order to work around these attitudes and prevent pregnant women from refusing the recommended treatment. This practice is frowned upon by many Western medical professionals who often take international precedence, as it is seen to jeopardize the right to informed consent for the patient. However, this is common practice as a necessity in some contexts when the diagnosis is upsetting or taboo, for example during terminal illness [21]. There is currently a code of ethics for medical practitioners in Sri Lanka [32] which emphasizes honestly, but previous studies have shown there is lacking awareness of this document among doctors [27]. Some scientists have argued that in some contexts, a white lie is sometimes the best professional option to achieve a therapeutic goal [24] which may be the case in this instance where people are unwilling to seek treatment.

The difficulty in helping mothers seek treatment in this community might be an effect of generally poor mental health knowledge in mothers, even among those who have received higher education. Midwives described themselves as the primary educators of their pregnant women and pregnant women’s families on mental health knowledge. More knowledge and changing attitudes towards mental health could promote health seeking behaviors of mothers, as suggested in a previous study with a Sri Lanka population [5]. More widespread knowledge and earlier detection may be key interventions to reduce the antenatal depression burden in this healthcare system, as has been demonstrated with other psychological disorders [18, 19].

Ideally, midwives suggested that the psychiatrist could make home visits in the same fashion that PHMs do, for better patient access and communication. However, there continues to be a shortage of psychiatric specialists in Sri Lanka, possibly leaving psychiatrists without time to commit to the family-based care midwives can conduct [17]. With further training in mental health, it is possible that midwives could take the role of community mental health workers during the antenatal period like in what has been done to reduce perinatal depression in other contexts [22, 33, 34]. Given the Bope Poddala region contains 18 midwives and the estimated population from the most recent census is 50331 people, each midwife is responsible for 2796 people, meeting the recommendations for a ratio below 5000 people per midwife in urban areas [10, 26]. Nonetheless, midwives may also not be able to take on additional duties, as they already report reduction in the quality of their care due to overwork, and frontline healthcare workers worldwide report a high rate of burnout [12].

## Limitations

We invited all 18 midwives in the study site but 6 midwives were not willing to participate due to unavailability or lack of interests. We did not collect the information of characteristics of the midwives who refused to participate, and thus we did not know if there were any defining differences between participants and non-participants. However, the 12 participants included are from all four clinics in the site, so we believe that the sampling bias would be limited. In addition, the core investigator (SW) who led data collection and analysis had limited field experience due to travel constraint caused by COVID-19 pandemic. The research team failed to collect non-verbal data in addition to the verbal recordings, which retracts from the richness of qualitative data. Data analysis also only took place after the data collection had finished, but we worked closely with the interviewers to ensure data trustworthiness. This data is only representative of the Bope Poddala division, and should not be used to draw conclusions about all practices across the diverse nation. The Bope Poddala division is unique in that it hosts an academic medical faculty, and therefore has access to special resources that other divisions do not. Because the of the psychiatric department at the medical faculty, referral to the psychiatrist is probably more common than it would be in other areas. In the future there should be further investigation on how clinical practice differs in other settings.

## Conclusions

This study is the first of its kind to internationally report on practices of public health midwives caring for people with antenatal depression in Sri Lanka. These findings are important not only to inform policy within Sri Lanka, but also to contribute to global knowledge of antenatal mental healthcare. Antenatal depression is a problem faced by many women in this community, and the most crucial barriers for effective care are the lack of recognition and standardization of the process. Public health midwives in the Bope Poddala division followed the clinical protocol to refer pregnant women who may need intervention for antennal depression and conduct follow-up visits during the treatment periods. However, the clinical guideline on the diagnosis of antenatal depression is lacking key details on symptoms for appropriate diagnosis. More official context-informed guidelines for effective practice and compulsory screening, much like what has been implemented for post-partum depression, is a crucial step towards providing high-quality, evidence-based care. Further research is needed to explore intervention strategies addressing current challenges to improving antenatal depression management in Sri Lanka.

## Supplementary Information

**Additional file 1:** Relevant text collected from treatment protocol.

## Data Availability

The data in the form of interviews generated during the current study are not publicly available in order to protect the privacy of the participants. The protocol analysed during the current study is available in the University of Kelaniya repository, https://medicine.kln.ac.lk/depts/publichealth/Fixed_Learning/clearkship/3.PHM/maternal_care_package_a_guide_to_field_healthcare_workers_english.pdf

## Abbreviations

PHM: Public Health Midwife
EPDS: Edinburgh Postpartum Depression Scale
MOH: Medical Officer of Health
