# Supplementary material for: Antenatal depression in Sri Lanka: a qualitative study of public health midwives’ views and practices
Source: Reprod Health. 2022 Jan 28;19:23. doi: 10.1186/s12978-022-01330-z (PMC8796187; doi:10.1186/s12978-022-01330-z)
Supplement: Supplementary file 1 — Additional file 1: Relevant text collected from treatment protocol. [file 12978_2022_1330_MOESM1_ESM.docx]

| Page | Ch. |  |
| --- | --- | --- |
|  |  | KEYWORD: depress* |
| 167 | 22 | Home Visit 3  Mother: Enquiries should be made about general wellbeing and all common health problems as described in first visit. All post natal women should be asked about resumption of sexual intercourse and possible dyspareunia as part of an assessment of overall well-being. Asses the mental condition of the mother – rest, sleep, unhappiness, loneliness All women should be asked about resolution of symptoms of maternal blues. If symptoms have not resolved, the woman’s psychological well-being should continue to be assessed for postnatal depression. Refer to MOH clinic. Continue to observe for any indication of domestic abuse. |
|  |  | KEYWORD: mental |
| 45 | 5 | Mental Disorders  Refer to a consultant Obstetrician. He/she will manage the case with other relevant specialists (psychiatrist/ MO mental health) |
| 46 | 6 | Home Visit 1  Assess the condition of the pregnant woman (physical, mental, social and environmental) |
| 50 | 6 | Advice, questions and answers, and scheduling the next appointment  Discuss with the woman and the family regarding the importance of maintaining the good mental status during pregnancy |
| 51 | 6 | Home Visit 2  Ask:  About her mental wellbeing (happiness, support from the family members) |
| 51 | 6 | Interventions and actions:  Educate mother and care givers regarding danger signals during pregnancy (antepartum haemorrhage, severe abdominal pain, severe headache), importance of clinic visits, proper nutrition, monitoring of weight gain, working and mental wellbeing during pregnancy, items to be taken to the hospital for the delivery, ECCD, rest and sleeping |
| 63 | 7 | Risk condition: Mental disorders  Frequency of home visits: Monthly home visits  Examine: Check compliance of drugs  Remarks: Educate on family members on the disease and advice on family support Ask family members to seek medical advice in case of a behavioural change of the mother Explain that drugs can be used even during breast feeding and not harmful for the baby Explain family members that mental disorders can be aggravated during post-partum period and to be more cautious Educate family members on postpartum psychological disorders |
| 89 | 11 | Assess and educate on factors other than dietary which could affect the maternal weight gain:  Assess the mental support she possess and educate regarding the association between maternal mental relaxation and fetal well being |
|  |  | KEYWORD: psych |
| 19 | 4 | Booking visits (6 – 8 weeks)  Objectives:  To assess the psychological/nutritional status and take necessary actions |
| 20 | 4 | Booking visits (6 – 8 weeks)  Medical history (past and present)  Specific diseases and conditions: tuberculosis, heart disease, chronic renal/hepatic disease, epilepsy, diabetes mellitus, hypertension, psychiatric illnesses and any chronic diseases |
| 23 | 4 | Booking visits (6 – 8 weeks)  Implement for following interventions  If woman is on treatment for diabetes, hypertension, epilepsy, psychiatric disease or any other disease refer them to the relevant clinics for continuous treatment |
| 45 | 5 | Mental disorders  Refer to a consultant Obstetrician. He/she will manage the case with other relevant specialists (psychiatrist/ MO mental health) |
| 63 | 7 | Risk condition: Mental disorders  Frequency of home visits: Monthly home visits  Examine: Check compliance of drugs  Remarks: Educate on family members on the disease and advice on family support Ask family members to seek medical advice in case of a behavioural change of the mother Explain that drugs can be used even during breast feeding and not harmful for the baby Explain family members that mental disorders can be aggravated during post-partum period and to be more cautious Educate family members on postpartum psychological disorders |
|  |  | KEYWORD: behaviour |
| 51 | 6 | Home visit 2 (22 – 24 weeks)  Examination:   - Assess the personnel hygiene - Observe any behavioural changes |
|  |  | Home visit 3 (34 – 36 weeks)  Examination:  - Assess personnel hygiene  - Observe for any behavioural changes |
| 63 | 7 | Risk condition: Mental disorders  Frequency of home visits: Monthly home visits  Examine: Check compliance of drugs  Remarks: Educate on family members on the disease and advice on family support Ask family members to seek medical advice in case of a behavioural change of the mother Explain that drugs can be used even during breast feeding and not harmful for the baby Explain family members that mental disorders can be aggravated during post-partum period and to be more cautious Educate family members on postpartum psychological disorders |
